# 15. Evaluation of Retained Immunity for *Tetanus-Diphtheria* and *Pneumococcal* Vaccines in Recipients of Cellular Therapies

**DOI:** 10.1093/ofid/ofab466.217

**Published:** 2021-12-04

**Authors:** Georgios Angelidakis, Roy F Chemaly, Partow Kebriaei, Nadim J Ajami, Micah M Bhatti, Elizabeth Shpall, Chitra Hosing, Preetesh Jain, Kris Michael Mahadeo, Fareed Khawaja, Jennifer Wargo, Robert Jenq, Ella Ariza Heredia

**Affiliations:** 1 : Departments of Infectious Diseases, Infection Control and Employee Health, houston, Texas; 2 The University of Texas MD Anderson Cancer Center, Houston, TX; 3 MD Anderson Cancer Center, Houston, Texas; 4 Laboratory Medicine, Houston, Texas; 5 University of Texas MD Anderson Cancer Center, Houston, Texas; 6 The University of Texas MD Anderson Cancer Center, Houston, Texas, Houston, TX

## Abstract

**Background:**

Infectious complications in cancer patients (pts) who have received T-cell therapies are similar to those in autologous hematopoietic stem cell transplant (HCT) recipients, who - because they lose prior acquired immunity after undergoing conditioning regimens and transplantation- may be at an increased risk for vaccine-preventable infections. We sought to determine seroprotection rates against pneumococcus and tetanus-diphtheria before and after cellular therapies.

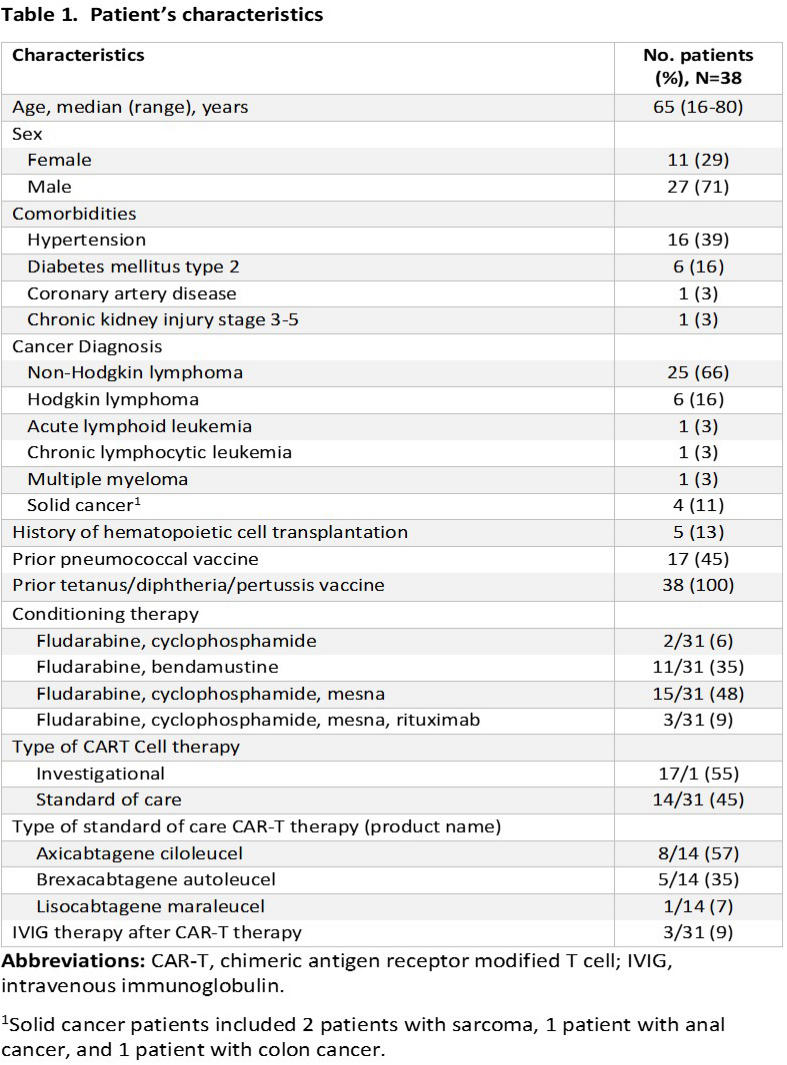

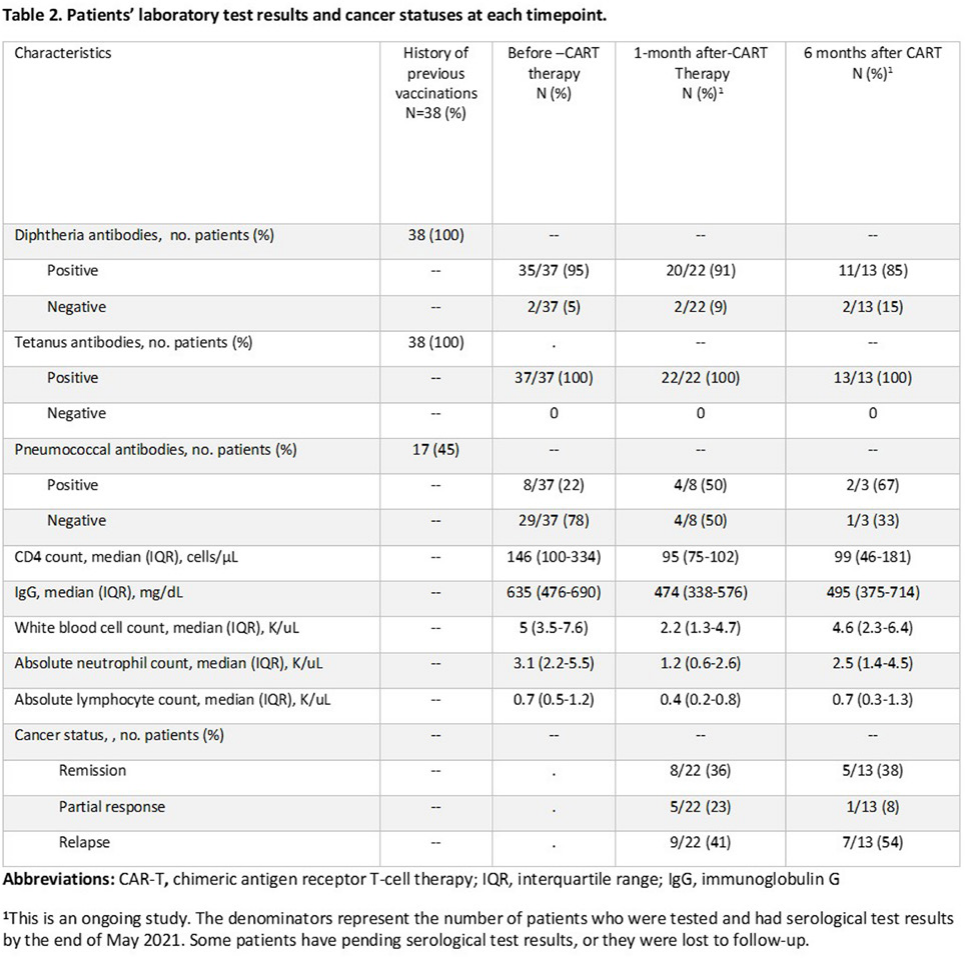

**Methods:**

In this ongoing prospective observational cohort study, we enrolled pts with any type of cancer who received cellular therapy with chimeric antigen receptor modified T cell (CAR-T), natural killer CAR-T, or T-cell receptor- directed immunotherapies at MD Anderson Cancer Center from January 2020 through May 2021. We performed antibody assays for diphtheria, tetanus, and pneumococcus before, at 1 month, and between 3-6 months after T-cell therapy for each pt regardless of vaccination history.

**Results:**

Of 38 pts enrolled, 27 (71%) were men and 25 (66%) had non-Hodgkin lymphoma (Table 1); 38 (100%) and 17 (45%) had a history of previous diphtheria-tetanus-acellular pertussis (Tdap) and pneumococcal vaccination, respectively (Table 2). Tetanus serologies were positive for all pts tested before, at 1 month and 3-6 months after T cell therapy (37/37 [100%], 22/22 [100%], and 13/13 [100%], respectively). Diphtheria serologies were positive for most pts tested before, at 1 month and 3-6 months after therapy (35/37 [95%], 20/22 [91%], and 11/13 [85%], respectively]. Pneumococcal serologies were positive for 8 out of 37 [22%] pts before therapy, among these 8 pts, 4 had positive serologies 1 month after therapy, and 2 of 3 tested 3-6 months after therapy had positive serologies. One pt received a pneumococcal vaccine 10 months after therapy but had negative serologies post-vaccination.

**Conclusion:**

Most pts who received T-cell therapy retained their immunity for diphtheria and tetanus, but most also lost their immunity for pneumococcus. This suggests that the standard of care for pts receiving T-cell therapy should include more robust strategy for pneumococcal vaccination, but its timing, need for booster dosing, and antibody response needs to be determined in future trials.

**Disclosures:**

**Roy F. Chemaly, MD, MPH, FACP, FIDSA**, **AiCuris** (Grant/Research Support)**Ansun Biopharma** (Consultant, Grant/Research Support)**Chimerix** (Consultant, Grant/Research Support)**Clinigen** (Consultant)**Genentech** (Consultant, Grant/Research Support)**Janssen** (Consultant, Grant/Research Support)**Karius** (Grant/Research Support)**Merck** (Consultant, Grant/Research Support)**Molecular Partners** (Consultant, Advisor or Review Panel member)**Novartis** (Grant/Research Support)**Oxford Immunotec** (Consultant, Grant/Research Support)**Partner Therapeutics** (Consultant)**Pulmotec** (Consultant, Grant/Research Support)**Shire/Takeda** (Consultant, Grant/Research Support)**Viracor** (Grant/Research Support)**Xenex** (Grant/Research Support) **Fareed Khawaja, MBBS**, **Eurofins Viracor** (Research Grant or Support) **Ella Ariza Heredia, MD**, **Merck** (Grant/Research Support)

